# *Mansonella perstans* microfilaremic individuals are characterized by enhanced type 2 helper T and regulatory T and B cell subsets and dampened systemic innate and adaptive immune responses

**DOI:** 10.1371/journal.pntd.0006184

**Published:** 2018-01-11

**Authors:** Manuel Ritter, Winston Patrick Chounna Ndongmo, Abdel Jelil Njouendou, Nora Nganyewo Nghochuzie, Lucy Cho Nchang, Dizzle Bita Tayong, Kathrin Arndts, Norman Nausch, Marc Jacobsen, Samuel Wanji, Laura E. Layland, Achim Hoerauf

**Affiliations:** 1 Institute of Medical Microbiology, Immunology and Parasitology, University Hospital of Bonn, Germany; 2 Parasite and Vector Research Unit (PAVRU), Department of Microbiology and Parasitology, University of Buea, Buea, Cameroon; 3 Research Foundation for Tropical Diseases and the Environment (REFOTDE), Buea, Cameroon; 4 Department of General Pediatrics, Neonatology, and Pediatric Cardiology, University Children’s Hospital, Medical Faculty, Duesseldorf, Germany; 5 German Centre for Infection Research (DZIF), partner site, Bonn-Cologne, Bonn, Germany; University of Liverpool, UNITED KINGDOM

## Abstract

The filarial nematode *Mansonella perstans* is endemic throughout Africa, northern South America and the Caribbean. Interestingly, *M*. *perstans*-infected individuals present no distinct clinical picture associated with certain pathology. Due to its relatively silent nature, research on this tropical disease has been neglected, especially *M*. *perstans*-driven immune responses. A hindrance in obtaining data on *M*. *perstans*-specific responses has been the inability to obtain adult worms since their habitats in serous cavities are difficult to access. Thus, in this study, for the first time, we used *Mansonella perstans* worm antigen extract as stimulant to obtain filarial-specific recall and immunoglobulin responses from *M*. *perstans* microfilaremic individuals (Mp MF+) from Cameroon. Moreover, systemic immune profiles in sera and immune cell composition in peripheral blood from Mp MF+ and amicrofilaremic individuals (Mp MF-) were obtained. Our data reveal that Mp MF+ individuals showed significantly reduced cytokine (IL-4, IL-6 and IL-12p70) and chemokine levels (IL-8 and RANTES), but significantly higher MIP-1β as well as increased *M*. *perstans*-specific IgG4 levels compared to Mp MF- individuals. In contrast, upon re-stimulation with worm antigen extract, IFN-γ, IL-13, IL-10 and IL-17A secretion was enhanced in cell cultures from Mp MF+ individuals when compared to those from cultures of healthy European individuals. Moreover, analysis of immune cell composition in peripheral blood from Mp MF+ individuals revealed increased type 2 helper T (Th2), natural killer (NK), regulatory B and T cell (Breg and Treg) subsets but decreased type 1 regulatory T (Tr1) cells. In summary, this study deciphers for the first time, *M*. *perstans*-specific immune responses using worm antigen extract and shows that patent *M*. *perstans* infections have distinct Th2, Breg and Treg subsets accompanied with reduced systemic innate and adaptive immune responses and dominant filarial-specific IgG4 levels.

## Author summary

Although there are approximately 114 million infected people with *Mansonella perstans*, there is a paucity of information about how this filarial nematode affects the host's immune system. *M*. *perstans* currently poses a potential risk to 581 million people throughout Africa and for many years infections were considered to be nonpathogenic because individuals presented undefined and non-distinct clinical symptoms. Due to lack of treatment and the potential consequences on co-infections, *M*. *perstans* infections are however, regarded as a public health problem in endemic areas. The long-lasting co-existence of the worm with its human host implies potential immune-modulatory properties that may also affect responses to other infections; an aspect that has not been well addressed. In this study, we were interested in determining whether *M*. *perstans*-infected patients from Cameroon show a distinct immunological profile. In addition, we applied for the first time *M*. *perstans* worm antigen extract in order to test its re-stimulation capacity on cells isolated from *M*. *perstans*-microfilaremic individuals. Overall this study shows that *M*. *perstans*-microfilaremic individuals have a distinct immune profile, which is characterized by increased Th2 and regulatory cell populations concomitant with reduced systemic cytokine/chemokine and increased filarial-specific IgG4 levels. In addition, the *M*. *perstans*-specific down-regulation of immune responses might be an explanation for the increased susceptibility and worsened disease course of HIV, tuberculosis and malaria in *M*. *perstans* endemic regions.

## Introduction

Worldwide approximately 240 million individuals are known to be infected with tropical threadlike nematodes from the family Onchocercidae and infections can persist for numerous years in man due to the helminth's immunomodulatory capacity on the host's immune system [1–7]. The well-adapted filarial helminth *Mansonella perstans* is endemic in tropical parts of Latin America and large proportions of Africa, with an estimated infection rate of 114 million people in over 33 countries [8]. Interestingly, unlike lymphatic filariasis (LF) or onchocerciasis, *M*. *perstans* infections cause only mild symptoms [8, 9]. Although, several clinical reports on subcutaneous swellings, abdominal pain, skin rashes, pericarditis and pleuritis have been linked to *M*. *perstans* infection [8], the abscence of a specific clinical condition has resulted in a shortfall about this filariae's suave evasion tactics. Research with *M*. *perstans* has also been hindered by the lack of specific antigen since adult worms reside in body cavities: the pericardium, the mesentery, and the perirenal and retroperitoneal connective tissues and are thus only rarely recovered [8–10]. As with other filariae, humans are infected through a bite of an insect vector; here the biting midge belong to the genus *Culicoides* [8]. However, in many endemic areas the *Culicoides* species that actually transmits *M*. *perstans* remains unclear. Although the exact chronology of *M*. *perstans* adult worm development remains ambiguous, fecund adult females release numerous microfilariae (MF), which circulate in the peripheral blood and can be taken up by another *Culicoides* biting midge to complete the cycle [8–10].

Interestingly, immunological studies on *M*. *perstans*-infected individuals have often been performed in those with filarial co-infections or other parasitic infections like hookworms, malaria or *Entamoeba* species [11–15]. Surprisingly, there is very little research on the immune responses during *M*. *perstans* mono-infection. In accordance with other filarial infections, *M*. *perstans* induce eosinophilia and increased IgE levels [16–18], but until now no study has investigated specific immune responses in *M*. *perstans-*infected individuals which are not co-infected with other filarial nematodes. Therefore, we compared systemic immune profiles in sera of *M*. *perstans* microfilaremic (Mp MF+) and amicrofilaremic (Mp MF-) individuals from Cameroon. Furthermore, we determined *M*. *perstans*-specific responses and immune cell populations in peripheral whole blood as well as *M*. *perstans*-specific IgG4 and IgE in sera. Our data revealed that with exception of IL-10 and MIP-1β, Mp MF+ individuals showed reduced serum levels of the chemokines IL-8 and RANTES and cytokines IL-4, IL-6 and IL-12p70. However, upon re-stimulation with *M*. *perstans* worm antigen extract, an increased production of IFN-γ, IL-10, IL-13 and IL-17A was detected in cultures stemming from Mp MF+ individuals when compared to those from healthy European individuals (non-endemic normals, NEN). Comparisons of total and *M*. *perstans*-specific IgG4 and IgE levels revealed higher filarial-specific ratio of filarial-specific IgG4/IgE in Mp MF+ individuals. In addition, specific immune cell populations in peripheral whole blood from Mp MF+ individuals consisted of increased CD4^+^CRTH2^+^ (Th2 cells), CD4^+^CD24^high^CD38^high^CD1d^high^ (regulatory B cells) and CD4^+^CD127^-^CD25^high^ (regulatory T cells) but reduced CD4+α/βTCR^+^CD49b^+^Lag-3^+^ (Tr1 cells) frequencies indicating that systemic cytokine/chemokine and immunoglobulin secretion is modulated by distinct regulatory immune cell populations in Mp MF+ individuals.

## Methods

### Study population and ethics

In 2015 and 2016, 16 *M*. *perstans*-microfilaremic individuals (Mp MF+) without visible pathology or health complaints and 38 *M*. *perstans* amicrofilaremic (Mp MF-) individuals were recruited from the health districts of Buea (Bomaka, Mile14, Minicokuette, Molyko and Sampit communities), Konye (Matondo, Baduma and Weme communities), Kumba (Ediki and Mbalangui communities) and Tombel (Mbule community) in the South-West region of Cameroon. An ethical clearance was obtained from the National Institutional Review board, Yaoundé (REF: N° 2015/09/639/CE/CNERSH/SP) and administrative clearance from the Delegation of Public Health, South-West region of Cameroon (Re: R11/MINSANTE/SWR/RDPH/PS/259/382). Approval for the study was granted by the "National Ethics Committee of Research for Human Health" in Cameroon. Enrolment into the study was done on a strictly voluntary basis and the objectives, risks and benefits of the study were explained in detail to all individuals [19]. In addition, blood samples from 4 NEN were also sampled from healthy European volunteers in Bonn that had never travelled to *M*. *perstans* endemic areas. Written informed consent and consent to use the data for research was obtained from all participants. Since two participants were minors the parents provided consent on their behalf. A detailed overview about the study population is provided in Table 1.

**Table 1:** Characteristics of the overall study population. According to their diagnostic status, individuals were categorized as either *M*. *perstans-*microfilaremic (Mp MF+) or amicrofilaremic (Mp MF-). Microfilariae (MF) numbers are given as mean and median with range. Four healthy European donors (non-endemic normals, NEN) were also recruited into the study. The table shows total sample size, age, gender, health districts and communities of the participants and numbers of individuals which are positive for Ov16-specific antibodies and soil-transmitted helminths (STHs).

Characteristics	Mp MF+	Mp MF-	NEN
Total sample size (n)	16	38	4
Mean age (range) [years]	37 (26–64)	35.5 (10–67)	44.5 (34–55)
Median age (range) [years]	34 (26–64)	34.5 (10–67)	44.5 (34–55)
Gender [female:male]	1:15	16:22	0:4
Health district	Konye, Kumba, Tombel	Konye, Kumba, Tombel, Buea	Bonn
Community	Baduma, Matondo, Mbalangui, Mbule	Baduma, Bomaka, Ediki, Matondo, Mbalangui, Mbule, Minicokuette, Molyko, Sampit, Mile-14	Bonn
Mean of microfilaria count (range) [MF/ml]	153 (1–745)	0	0
Median of microfilaria count (range) [MF/ml]	70 (1–745)	0	0
Number of Ov16-specific IgG4 positive individuals	12 (out of 15)	15 (out of 28)	0
Number of individuals positive for STHs	2 (*Ascaris lumbricoides*) 1 (*Trichuris trichiura*)	0	0

### Parasitic assessment

All participants were asked to participate in answering a questionnaire to assess current and/or previous infections and treatments. *M*. *perstans* infections were diagnosed via thick blood film, Sedgewick rafter counting and filtration techniques. For the thick blood film technique, peripheral whole blood was applied on a glass slide, stained with Giemsa and examined for microfilariae (MF) under the microscope at x10 magnification as described previously [20]. In addition, 100μl whole blood was mixed with 900μl of 3% acetic acid, poured onto a Sedgewick rafter counting chamber (VWR, Langenfeld, Germany) and MF counts were examined using a microscope at x10 magnification. For the filtration technique, a 3μm Whatman nucleopore membrane filter (Sigma-Aldrich, Munich, Germany) was placed between filter holders. Using a syringe, 1ml of whole blood was filtered through the Whatman membrane, flushed with 4-5ml distilled water to wash off the remaining blood and fixed with 1ml methanol. Finally, the membrane was placed on a glass slide, stained with Giemsa and examined for MF at x10 magnification. The average of the MF counts from the three different methods was then calculated to discriminate *M*. *perstans* microfilaremic (Mp MF+) individuals. To exclude active co-infections, individuals provided two skin snips and were visually investigated for nodules to rule out *Onchocerca volvulus* (*O*. *volvulus*) infections [21, 22] and additionally, thick blood smears were prepared for the detection of *Loa loa* or *Plasmodium* infection. Since the prevalence of *Wuchereria bancrofti* (*W*. *bancrofti*) is under 1% in the targeted health districts [23], infections with this filariae were not specifically determined (no night blood sampling) but still checked for on individual slides and no infections were found. In addition, serum samples were tested for IgG4 antibodies against *O*. *volvulus* Ov16 and *W*. *bancrofti* Wb123 antigens using the SD BIOLINE Oncho/LF IgG4 biplex test (Alere, Cologne, Germany; Distributor in Cameroon) according to manufacturer’s instructions. This test have a sensitivity rate of >90% and a specificity rate of >98% [24]. Gastrointestinal infections were detected by Kato-Katz on stool samples and only three Mp MF+ individuals had further *Ascaris* or *Trichuris* infections. No infections with schistosomes were detected. Detailed information about the study population and their parasitic infections are shown in Table 1.

### Cytokine and chemokine analysis of serum samples

Cytokine and chemokine levels from serum samples of study participants in the endemic areas were determined using a ProcartaPlex Human Cytokine/Chemokine/Growth Factor Panel 1 (eBioscience, Frankfurt, Germany) according to the manufacturer’s instructions. Serum samples were 1:2 diluted in PBS. The detection limit depends on the individual analyte but is between 0.016–0.237pg/ml according to the manufactures description. Data were acquired using a MAGPIX Luminex system (Luminex Cooperation, Austin, USA) and analyzed with ProcartaPlex Analyst software 1.0 (eBioscience).

### *M*. *perstans in vitro* culture and preparation of worm antigen extract

To investigate the development of *M*. *perstans* life stages, we established a culture system to maintain worms *in vitro* as previously described [25]. In brief, *M*. *perstans* infective larvae (L3) were obtained from *Culicoides* midges following a blood meal on a consenting donor who had a high peripheral MF load. Midges were captured and kept for 12 days at 23°C, the time required for the development of L3 from MF in the vector. After 12 days, L3 were isolated from the midges and cultured in Dulbecco’s Modified Eagle Medium (DMEM; Thermo Fisher Scientific, Schwerte, Germany) supplemented with 10% foetal bovine serum (FBS; Lonza, Verviers, Belgium) containing a confluent monolayer of monkey kidney epithelial cells (LLC-MK2; LGC Standard GmbH, Wesel, Germany). Infective larvae were cultured for 50 days at 37°C and viability, growth and moulting were assessed on a daily basis via microscopy. From this *in vitro* culture, 52 male and female *M*. *perstans* worms (L5 larvae) were mechanically minced on ice in cold sterile endotoxin-free PBS. Insoluble material was removed by centrifugation at 300g for 10min at 4°C. The protein concentration of the *M*. *perstans* worm antigen extract was determined using the Advanced Protein Assay (Cytoskeleton, ORT, USA). Aliquots were frozen at -80°C until required. To verify the absence of bacterial contamination, *M*. *perstans* antigen preparation was plated onto a BD BBL Columbia CNA agar plates (BD, Heidelberg, Germany) and incubated for 72h at 37°C. No bacterial growth was determined. Furthermore, levels of endotoxin in the worm antigen extract were measured using the Pierce LAL Chromogenic Endotoxin Quantitation Kit (Thermo Fisher Scientific, Schwerte, Germany) and were under 0.1EU/ml.

### *In vitro* re-stimulation assays with *M*. *perstans* worm antigen extract

100μl whole blood from *M*. *perstans*-infected individuals and NEN were plated onto 96-well culture plates (Greiner Bio-One GmbH, Frickenhausen, Germany) and cultivated in 100μl RPMI-1640 medium (Sigma-Aldrich, Munich, Germany) including 10% bovine calf serum (BCS, Sigma-Aldrich). Whole blood cultures were then left un-stimulated or re-stimulated with 50μg/ml *M*. *perstans* worm extract for 72h at 37°C and 5% CO_2_. Thereafter culture supernatants were removed and frozen at -20°C until cytokine levels were determined by ELISA.

### Cytokine analysis of *M*. *perstans*-specific re-stimulation assays

Levels of IFN-γ, IL-4, IL-5, IL-10, IL-13 and IL-17A from the *in vitro* cell cultures were determined using the Ready-Set-Go ELISA kits (eBioscience, Frankfurt, Germany). The ELISAs were performed according to the manufacturer’s instructions. Absorbance levels were measured using the SpectraMAX ELISA reader (Molecular Devices, Sunnyvale, USA) with wavelength correction (450 nm and 570 nm). Data were analyzed with SOFTmax Pro 3.0 software (Molecular Devices).

### Assessment of immunoglobulins in sera

Levels of *M*. *perstans*-specific IgG4 and IgE were measured in individual serum samples using an extract of *M*. *perstans* worms following an established protocol for other filarial-specific Ig measurements [21, 26]. In brief, 96-well polysorb plates (Thermo Fisher Scientific) were coated overnight at 4°C with 2.5μg/ml *M*. *perstans* extract in PBS (pH 9.6). After 3 washes with 0.05% Tween/PBS (pH 7.2) and once with PBS alone, plates were blocked with 200μl/well of 1% BSA/PBS for two hours at room temperature. Following further washing, 50μl/well of diluted sera was added (1:500 for IgG4 and 1:20 for IgE) and incubated overnight at 4°C. After washing, 50μl/well of biotinylated secondary antibodies (IgG4 (1:15000) or IgE (1:1000)) were added for two hours at room temperature and after another wash step streptavidin-peroxidase (50μl/well; 1:5000) was added for 45 minutes at room temperature (Roche Diagnostics, Mannheim, Germany). After the final wash, reactions were developed with TMB (tetramethylbenzidine, 50μl/well), (Sigma-Aldrich) and stopped with 25μl/well of 2N H_2_SO_4_ (Sigma-Aldrich). Following the manufacturer's instructions, total IgE and IgG4 levels were measured using specific kits purchased from eBioscience (Human IgE/IgG4 Ready-SET-Go). Absorbance levels and data analysis were performed as described above.

### Analysis of immune cell composition in peripheral whole blood

In Cameroon, red blood cells were eliminated from peripheral whole blood using red blood cell lysis buffer (Sigma-Aldrich). Cells were then fixed and permeabilized with Fixation Buffer and Intracellular Staining Permeabilization Wash Buffer (Biolegend, San Diego, USA) according to the manufacturer’s instructions. Thereafter, cells were stained with combinations of fluorophores (FITC, Alexa Fluor 488, Alexa Fluor 647 PE, PE-Cy7, PerCp-Cy5.5, APC)-conjugated with anti-human CD3 (clone HIT3a), CD4 (clone RPTA-4), CD8a (clone HIT8a), CD16 (clone 3G8), CD56 (clone HCD56), CD152 (CTLA-4; clone L3D10), CD183 (CXCR3; clone G025H7), CD223 (LAG3; clone C9B7W), CD294 (CRTH2; clone BM16), α/βTCR (cloneIP26) from Biolegend, CD127 (clone eBioRDR5) from eBioscience, CD1d (clone CD1d42), CD19 (clone HIB19), CD24 (clone ML9), CD25 (clone M-A251), CD49b (clone 12F1), CD279 (PD-1; clone MIH4) from BD Bioscience (Heidelberg, Germany) or CD161 (clone 191B8) from Miltenyi Biotec GmbH (Bergisch Gladbach, Germany) monoclonal antibodies to determine distinct cell populations. Expression levels were determined using the BD Accuri flow cytometer (BD Bioscience) and analysed with the FlowJo v10 software (FlowJo, LLC, USA).

### Statistical analysis

Statistical analyses were performed using the software SPSS (IBM SPSS Statistics 22; Armonk, NY) and the PRISM 5 programme (GraphPad Software, Inc., La Jolla, USA). Variables did not meet assumption to allow parametric analysis, therefore to compare more than two groups a Kruskal-Wallis-test was performed and, if significant, followed by a Dunn`s multiple comparison test for a further comparison of the groups. In addition, the Mann-Whitney-U-test was used to compare two groups. The Spearman`s rank correlation coefficient was applied to analyse rank correlations between two variables and stepwise multiple logistic regression analysis was performed to decipher the influence of occult *O*. *volvulus* infection on the immunological results. P-values of 0.05 or less were considered significant.

## Results

### Study population

To characterize immunological responses during *M*. *perstans* infection, individuals were recruited between 2015 and 2016 from the health districts of Buea, Konye, Kumba and Tombel in the South-West region of Cameroon which are prevalent for *M*. *perstans* and the recruitment of individuals was based on the presence of *M*. *perstans* microfilariae (MF) in peripheral blood. To exclude co-infections, all individuals were screened for other filarial infections as well as for *Plasmodium* and schistosome infections. Only three *M*. *perstans* microfilaremic (Mp MF+) individuals were additionally infected with *Trichuris* or *Ascaris* and no schistosome or *Plasmodium* infection was detected. Individuals who were positive for *O*. *volvulus*, *Loa loa* or *W*. *bancrofti* MF (active infections) were not included within this study. In addition, serum from 15 Mp MF+ and 28 amicrofilaremic (Mp MF-) individuals were tested for IgG4 antibodies against Ov16 and Wb123 antigens to decipher exposure or occult infections with *O*. *volvulus* and *W*. *bancrofti*, respectively. Interestingly, 12 Mp MF+ and 15 Mp MF- individuals tested positive for IgG4 antibodies against Ov16 showing that individuals from both cohorts were exposed to *O*. *volvulus*. None of the tested individuals reacted positive against the Wb123 antigen which is in accordance with the low prevalence of this infection in the targeted health districts [23]. Mp MF+ individuals had a mean of 153, a median of 70 and a range of 1–745 MF/ml. The median age for both Mp MF+ and Mp MF- individuals was 34 and 34.5, respectively. Whole blood samples from four healthy European NEN individuals were collected as controls to test the specificity of the *M*. *perstans* worm antigen extract since these individuals have never been exposed to or been in an area endemic for *M*. *perstans*. An overview about the characteristics of the entire study population is depicted in Table 1. Due to the lack of serum or blood samples from some patients and the limited amount of *M*. *perstans* worm antigen extract, it was not possible to measure all parameters for all individuals. Moreover, due to the installation of a flow cytometer machine in Buea, additional amounts of fresh peripheral blood samples were obtained from a proportion of participants to decipher immune cell composition via flow cytometry. Additional information about the study population of the different experiments is depicted in the supporting information section (S1–S4 Tables).

### Decreased systemic levels of IL-4 and IL-17A in *M*. *perstans* microfilaremic individuals

**Fig 1:**
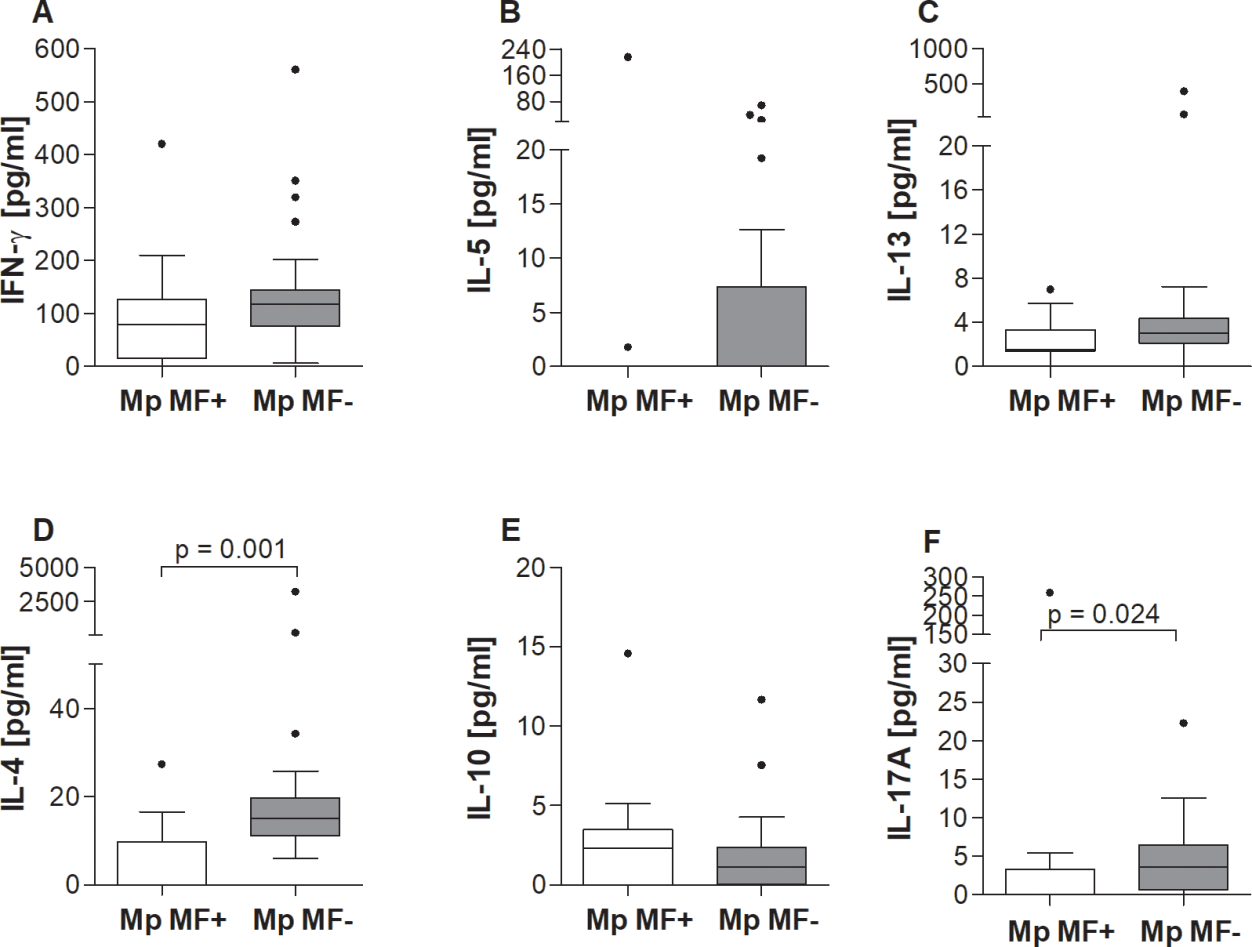
Reduced systemic IL-13, IL-4 and IL-17A levels in *M*. *perstans* microfilaremic individuals. Sera from *M*. *perstans*-microfilaremic (Mp MF+, n = 11) and amicrofilaremic (Mp MF-; n = 28) individuals were analyzed for the contents of (**A**) IFN-γ, (**B**) IL-5, (**C**) IL-13, (**D**) IL-4, (**E**) IL-10 and (**F**) IL-17A using luminex technology. Graphs show box whiskers with median, interquartile ranges and outliers. Statistical significances between the indicated groups were obtained using the Mann-Whitney-U-tests.

To survey the basic immunological milieu in Mp MF+ and Mp MF- individuals (S1 Table), sufficient IFN-γ, IL-5, IL-4, IL-13, IL-10 and IL-17A cytokine levels were analysed in serum samples (Fig 1A–1F). No significant differences could be observed between Mp MF- and Mp MF+ individuals with regards to IFN-γ, IL-5 or IL-13 (Fig 1A–1C). Systemic levels of IL-4 however were significantly lower in microfilaremic participants (Fig 1D). Interestingly, whereas IL-10 levels were comparable between the groups (Fig 1E), systemic IL-17A levels were markedly lower in *M*. *perstans*-microfilaremic individuals. Further analysis revealed negative correlations between MF and IL-4 (r = -0.512, p = 0.001), IL-13 (r = -0.325 p = 0.044) and IL-17A (r = -0.364 p = 0.023) but not with the other parameters. Although unintentional, there was a bias in gender towards women within the Mp MF- group which might influence the findings shown in Fig 1. Thus, we performed additional analyses in which we excluded women. Interestingly, with the exception of IL-17A, in which the significance was lost, the differences between the cytokine levels of the Mp MF+ and Mp MF- individuals remained unchanged (S1 Fig), showing that the observed cytokine secretion patterns were not influenced solely by gender.

### Elevated systemic MIP-1β but lower IL-6, TNF-α, IL-12p70, IL-8 and RANTES levels in *M*. *perstans* microfilaremic individuals

**Fig 2:**
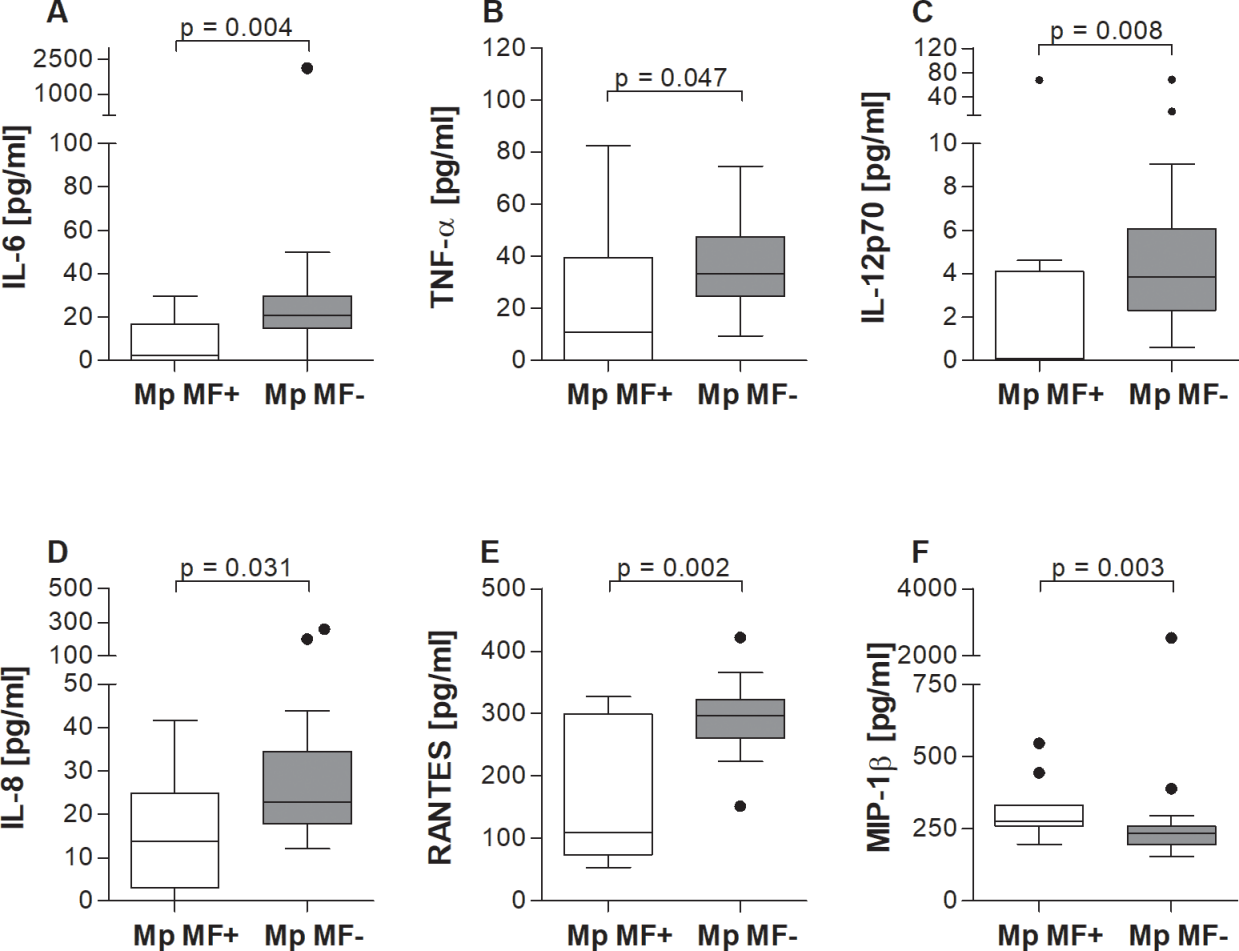
Elevated systemic levels of MIP-1β levels in *M*. *perstans* microfilaremic individuals. Sera from *M*. *perstans* microfilaremic (Mp MF+, n = 11) and amicrofilaremic (Mp MF-; n = 28) individuals were analyzed for the contents of (**A**) IL-6, (**B**) TNF-α, (**C**) IL-12p70, (**D**) IL-8, (**E**) RANTES and (**F**) MIP-1β using luminex technology. Graphs show box whiskers with median, interquartile ranges and outliers. Statistical significances between the indicated groups were obtained using the Mann-Whitney-U-tests.

Next, we measured levels of innate cytokines and chemokines in the serum of Mp MF- and Mp MF+ individuals. With regards to innate-associated cytokines, Mp MF+ individuals presented significantly lower levels of IL-6 (Fig 2A), TNF-α (Fig 2B) and IL-12p70 (Fig 2C). Amounts of systemic IL-6, TNF-α and IL-12p70 levels negatively correlated with MF load (r = -0.467, p = 0.003; r = -0.317, p = 0.049 and r = -0.433, p = 0.006 respectively). Mp MF+ individuals also presented significantly lower levels of IL-8 and RANTES (Fig 2D and 2E respectively) which also negatively correlated with MF counts (IL-8: r = -0.343, p = 0.033 and RANTES: r = -0.457, p = 0.003). However, systemic levels of MIP-1β were significantly higher in Mp MF+ individuals (Fig 2F) which further positively correlated with peripheral MF counts (r = 0.516, p = 0.001). Moreover, with the exception of TNF-α, exclusion of women did not alter the differences in the cytokine/chemokine levels between Mp MF+ and Mp MF- individuals (S2 Fig), again, showing that the observed cytokine and chemokine secretion patterns were not influenced by gender. In addition, since Mp MF- and Mp MF+ individuals tested positive for IgG4 antibodies against the Ov16 antigen, we determined if there were any associations between the presence of this *O*. *volvulus*-specific IgG4 antibody and the obtained immunological results using stepwise multiple logistic regression analysis. Interestingly, RANTES was the only correlate to show a significant association (p = 0.035) with *O*. *volvulus*-specific IgG4 antibodies. This suggests that the active *M*. *perstans* infection modulates the down-regulation of systemic immune responses.

### Higher ratio of filarial-specific IgG4 levels in *M*. *perstans* microfilaremic individuals

**Fig 3:**
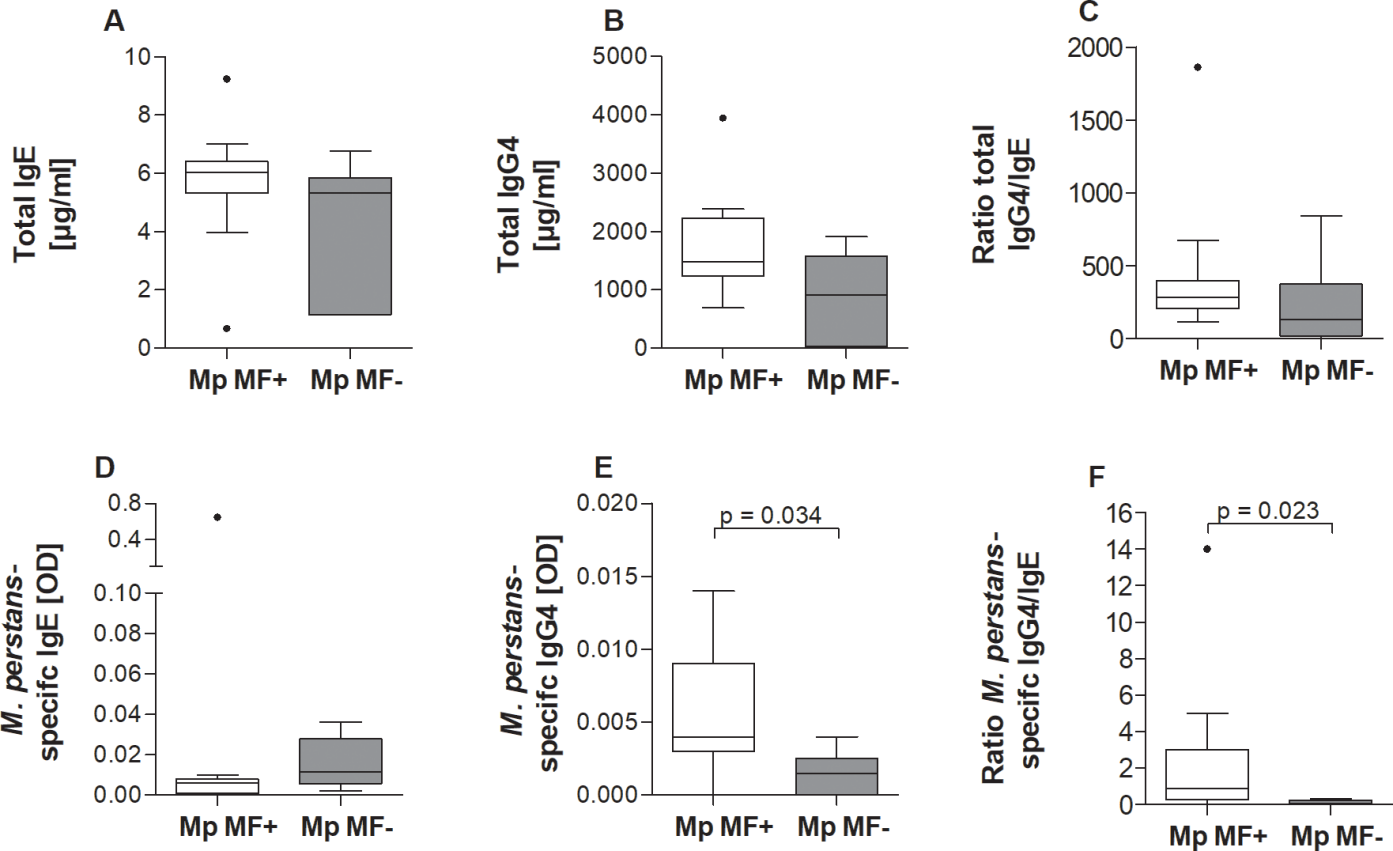
*M*. *perstans* microfilaremic individuals present elevated filarial-specific IgG4 in sera. Sera from and *M*. *perstans* microfilaremic (Mp MF+, n = 11) and amicrofilaremic (Mp MF-; n = 6) individuals were analyzed for total and *M*. *perstans*-specific immunoglobulin levels. (**A**) total IgE (μg/ml) (**B**) total IgG4 (μg/ml), (**C**) ratio of total IgE/IgG4, (**D**) *M*. *perstans*-specific IgE (OD), (**E**) *M*. *perstans*-specific IgG4 (OD) and (**F**) ratio of *M*. *perstans*-specific IgG4/IgE. Graphs show box whiskers with median, interquartile ranges and outliers. Statistical significances between the indicated groups were obtained using the Mann-Whitney-U-test.

In onchocerciasis and lymphatic filariasis, infected individuals often present a regulatory milieu with pronounced levels of IL-10, TGF-β, regulatory T cells and IgG4 being associated with high worm burden [1, 21, 26–29]. Thus, serum from Mp MF- and Mp MF+ individuals were analysed for immunoglobulin levels but due to the lack of *M*. *perstans* antigen only a portion of individuals shown in S1 Table were randomly selected (S2 Table). Nevertheless, upon comparison of total IgE, IgG4 and the ratio of total IgG4/IgE no differences were observed between the groups (Fig 3A–3C). Interestingly, we established an *in vitro* culture of viable *M*. *perstans* adult worms [25] which then were used to prepare worm antigen extract for the assessment of *M*. *perstans*-specific responses. Based on previous research [21, 26], we established an *M*. *perstans*-specific ELISA for detecting IgG4 and IgE and performed an initial screening of antibody levels in a smaller cohort of serum samples from Mp MF+ and Mp MF- individuals. Whereas *M*. *perstans*-specific IgE levels were slightly increased in Mp MF- individuals (Fig 3D), Mp MF+ individuals presented significantly higher *M*. *perstans*-specific IgG4 levels (Fig 3E). This phenotype was further reflected in the significantly higher *M*. *perstans*-specific IgG4/IgE ratio (Fig 3F). Again, the findings remained consistent when only men were retained within the analysis (S3 Fig).

### *M*. *perstans*-derived worm antigen extract provokes strong IL-10, IFN-γ, IL-13 and IL-17A responses in cultures of peripheral whole blood cells from *M*. *perstans* microfilaremic individuals

**Fig 4:**
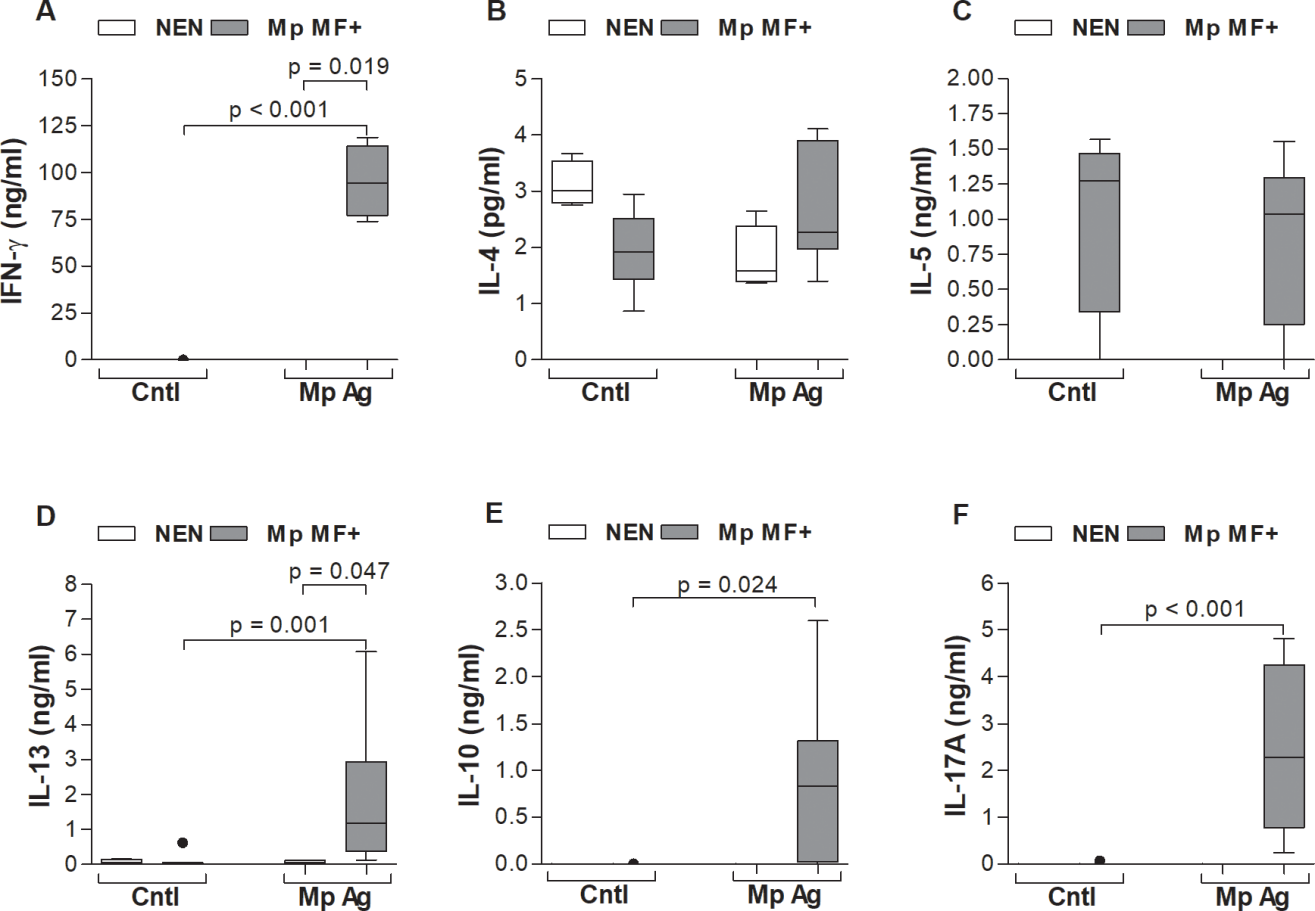
Dominant *M*. *perstans*-specific IL-10, IFN-γ, IL-13 and IL-17A production by peripheral cells from *M*. *perstans* microfilaremic individuals. Freshly isolated peripheral whole blood cells (100μl/well) from *M*. *perstans* microfilaremic individuals (Mp MF+; n = 9) and healthy European non-endemic normals (NEN; n = 4) were cultivated in 10% BCS/RPMI-1640 medium (100μl/well) and left either un-stimulated (Cntl) or cultured with *M*. *perstans*-derived worm antigen extract (Mp Ag, 50μg/ml) at 37°C for 72 hours. Thereafter, culture supernatants were analysed for levels of (**A**) IFN-γ, (**B**) IL-4, (**C**) IL-5 (**D**) IL-13, (**E**) IL-10 and (**F**) IL-17A by ELISA. Graphs show box whiskers with median, interquartile ranges and outliers. Statistical significances between the indicated groups were obtained using the Kruskal-Wallis-test and, if significant, followed by a Dunn`s multiple comparison test for further comparison of the groups.

To determine the ability of *M*. *perstans* worm antigen extract [25] to elicit *M*. *perstans*-specific responses we stimulated freshly isolated peripheral whole blood cells from Mp MF+ individuals and compared them to responses from healthy European NEN volunteers *in vitro*. The selection of NEN for this proof of principle work was to ensure that the responses observed from Mp MF+ individuals were specific since Mp MF- individuals might have had previous exposure to *M*. *perstans* and therefore also elicit responses. In addition, due to the limitation of *M*. *perstans* worm antigen extract only 4 NEN and 9 Mp MF+ individuals (S3 Table) could be included within the *in vitro* stimulation assay. After 72 hours, levels of IFN-γ, IL-5, IL-13, IL-4, IL-10 and IL-17A (Fig 4A–4F) were measured in the culture supernatant by ELISA. Whereas *M*. *perstans*-derived worm antigen extract provoked strong IFN-γ secretion from cultures of Mp MF+ individuals, none was elicited in cultures of NEN volunteers (Fig 4A). Levels of IL-4 were under the detection limit of the ELISA (below 5pg/ml) in both groups (Fig 4B). Levels of IL-5 were measurable in cultures of Mp MF+ individuals but remained comparable between unstimulated wells (control, Cntl) upon culture with *M*. *perstans-*derived worm antigen extract (Mp Ag; Fig 4C). In general, minimal levels of IL-5 or IL-13 were elicited in cultures from NEN with *M*. *perstans*-derived worm antigen extract (Fig 4C and 4D respectively), but strong IL-13 responses were provoked in whole blood cells from Mp MF+ individuals (Fig 4D). IL-10 secretion was also only observed in cultures of Mp MF+ individuals with *M*. *perstans*-derived worm antigen extract (Fig 4E). Besides IFN-γ, one of the strongest responses was the secretion of IL-17A in cultures of Mp MF+ individuals; no IL-17A was released in cultures of NEN volunteers (Fig 4F).

### Increased type 2 helper T cell (Th2) frequencies in *M*. *perstans* microfilaremic individuals

**Fig 5:**
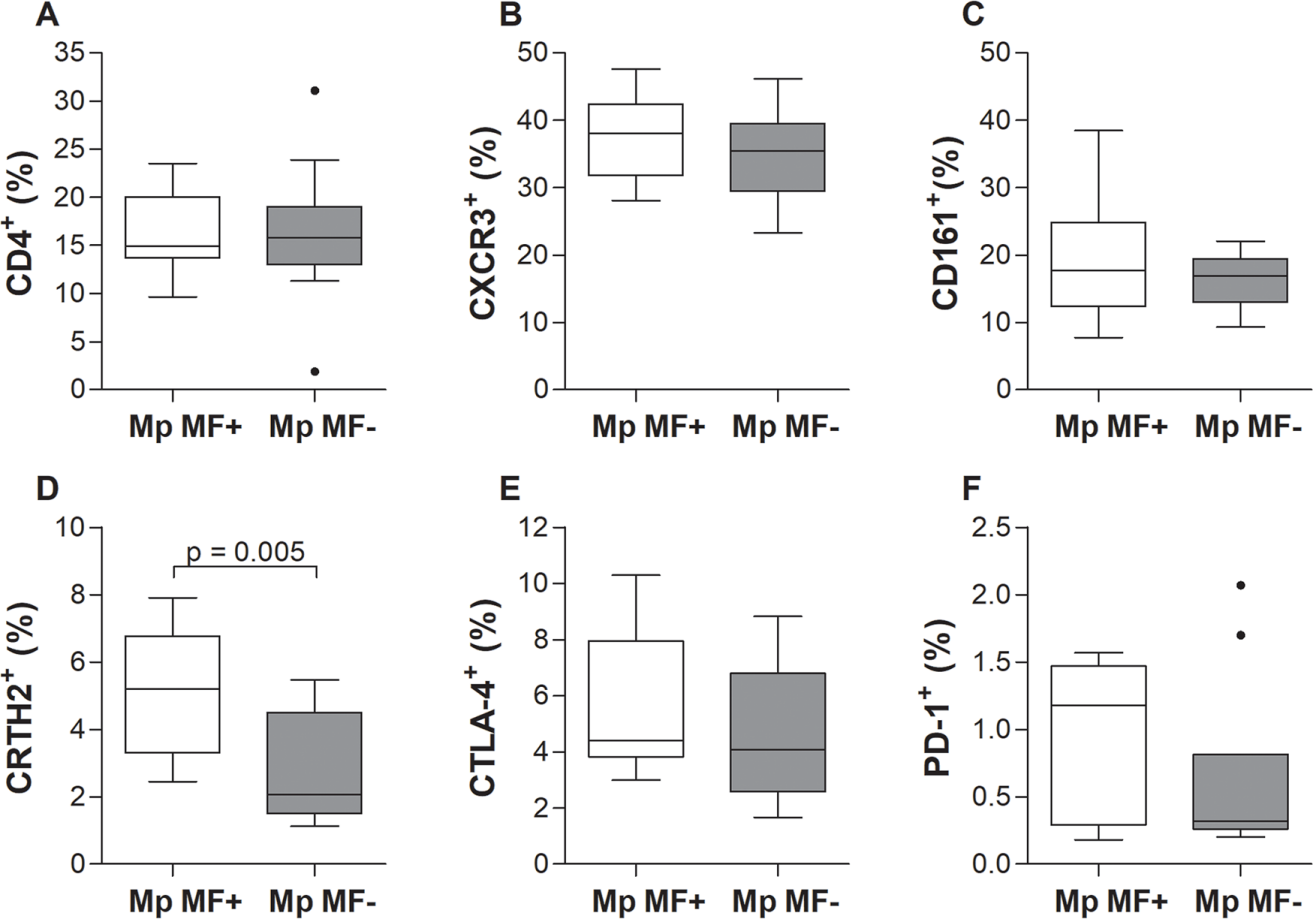
Increased CD4^+^CRTH2^+^ Th2 cell frequencies in peripheral blood of *M*. *perstans* microfilaremic individuals. Using flow cytometry, peripheral whole blood cells from *M*. *perstans* microfilaremic (Mp MF+; n = 11) and amicrofilaremic (Mp MF-; n = 10) individuals were analyzed for frequencies (%) of (**A**) CD4^+^ T cells on lymphocytes and CD4^+^ T cells expressing (**B**) CXCR3, (**C**) CD161, (**D**) CRTH2, (**E**) CTLA-4 or (**F**) PD-1. Graphs show box whiskers with median, interquartile ranges and outliers. Statistical significances between the indicated groups were obtained using the Mann-Whitney-U-test.

Since 2016 a flow cytometry machine was installed in Buea and therefore additional whole blood samples were obtained from the participants (S4 Table) to decipher immune cell populations which might be responsible for the observed cytokine, chemokine and immunoglobulins secretion patterns. Frequencies of CD4^+^ (Fig 5) and CD8a^+^ T cell populations (S4 Fig) were analysed in Mp MF+ and Mp MF- individuals according to the applied gating strategy (S5 Fig). Frequencies of CD4^+^ and CD8a^+^ T cells were comparable in Mp MF+ and Mp MF- individuals (Fig 5A and S4A Fig, respectively) and moreover no differences were determined in CD4^+^ T cells expressing CXCR3 (CD183) or CD161 which are markers for Th1 or Th17 cell populations (Fig 5B and 5C). In contrast, the frequencies of CD4^+^CRTH2^+^ (CD4^+^CD294^+^) Th2 cells were significantly higher in the Mp MF+ cohort (Fig 5D). Expression levels of the T cell activation markers CTLA-4 (CD152) and PD-1 (CD279) on CD4^+^ T cells were also equal between both groups (Fig 5E and 5F, respectively). Interestingly, whereas CTLA-4 expression on CD8a^+^ cytotoxic T cells was comparable between the groups (S4B Fig), expression levels of PD-1 were increased on cells from Mp MF+ individuals (S4C Fig).

### Augmented natural killer cell frequencies in *M*. *perstans* microfilaremic individuals

**Fig 6:**
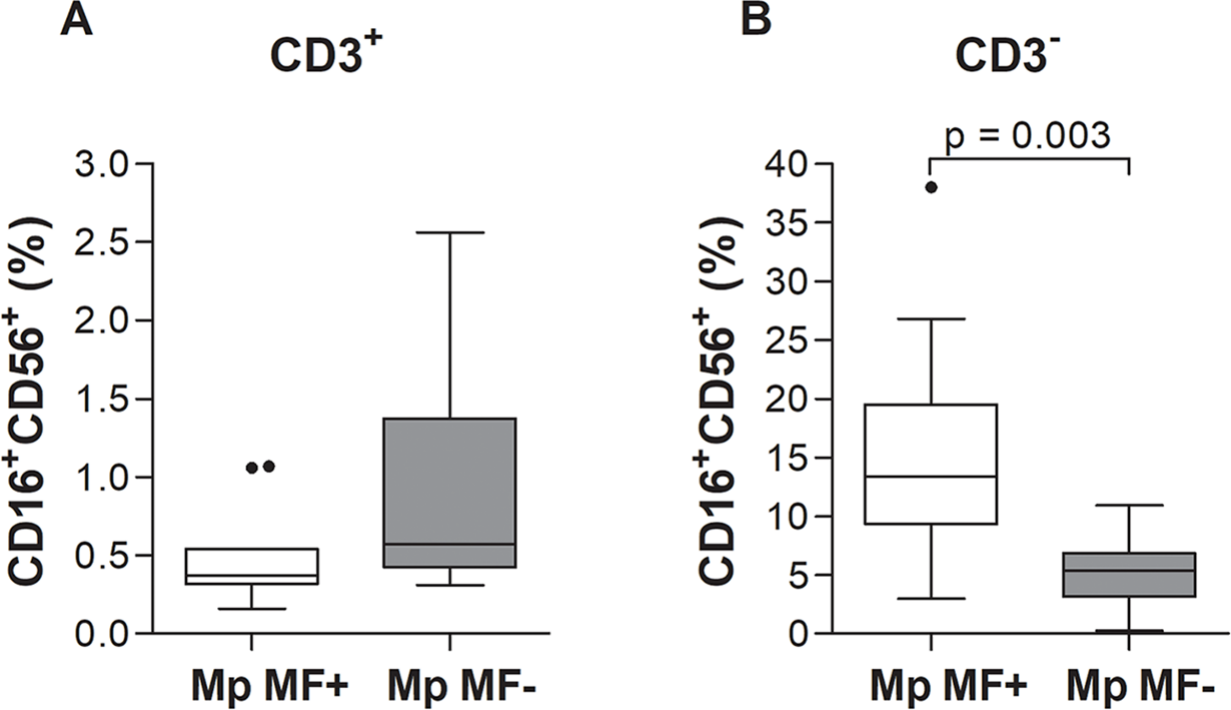
Elevated frequencies of CD3^-^CD16^+^CD56^+^ natural killer cells in peripheral blood of *M*. *perstans*-microfilaremic individuals. Using flow cytometry, peripheral whole blood cells from *M*. *perstans* microfilaremic (Mp MF+; n = 11) and amicrofilaremic (Mp MF-; n = 10) individuals were analyzed for frequencies (%) of (**A**) CD3^+^ T cells co-expressing CD16 and CD56 for NKT cells and (**B**) CD3^-^ cells co-expressing CD16 and CD56 to determine NK populations. Graphs show box whiskers with median, interquartile ranges and outliers. Statistical significances between the indicated groups were obtained using the Mann-Whitney-U-test.

Besides Th cells and cytotoxic T cells, other immune cell populations were shown to play a role during helminth infections, such as natural killer T (NKT) cells [1, 30]. Thus, we analysed NKT cell populations (CD3^+^CD16^+^CD56^+^; Fig 6A) in peripheral blood from Mp MF+ and Mp MF- individuals according to the applied gating strategy (S6 Fig) and observed comparable cell frequencies between the groups. However, frequencies of CD3^-^CD16^+^CD56^+^ natural killer (NK) cells were significantly increased in Mp MF+ individuals (Fig 6B), suggesting that *M*. *perstans* infection also influences the composition of innate immune cells.

### Elevated regulatory B and T cell subsets, but lower Tr1 cell frequencies in *M*. *perstans* microfilaremic individuals

**Fig 7:**
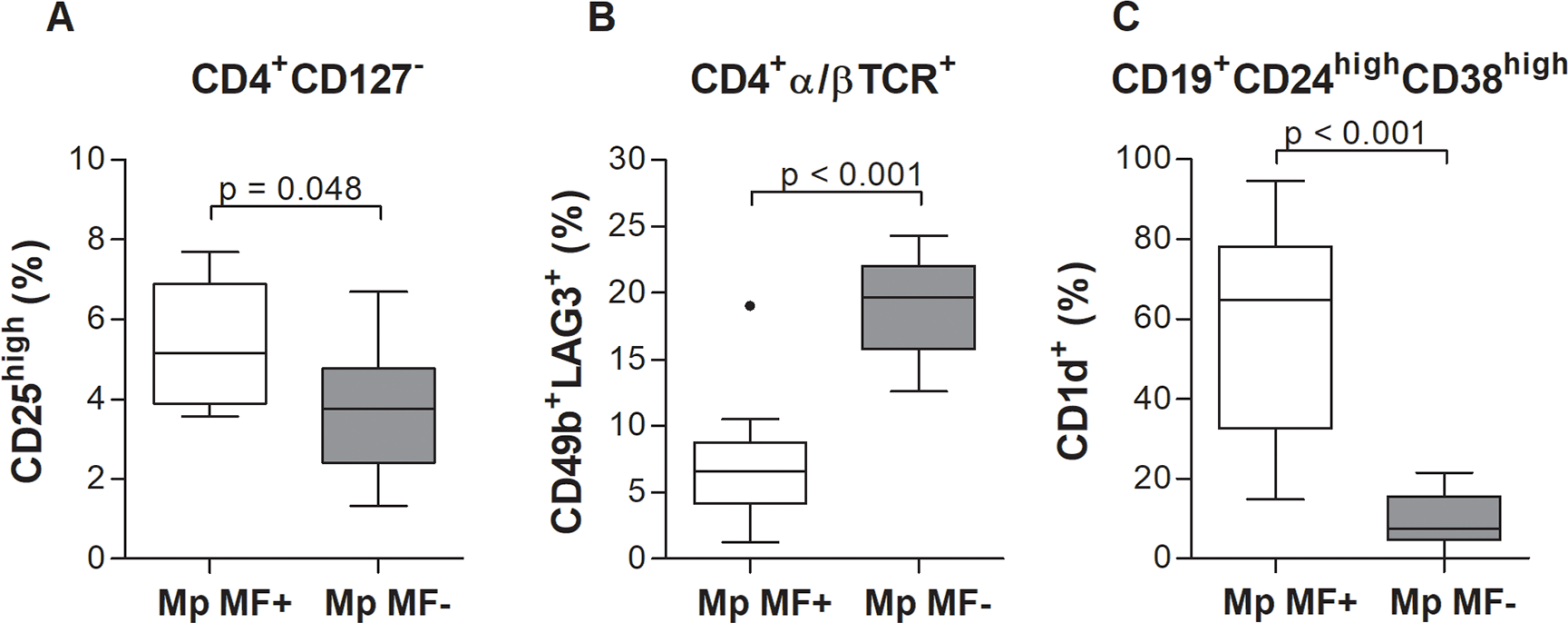
Increased frequencies of regulatory T and B cells (Tregs and Bregs) but decreased type 1 regulatory T (Tr1) cell populations in *M*. *perstans* microfilaremic individuals. Using flow cytometry, peripheral whole blood cells from *M*. *perstans* microfilaremic (Mp MF+; n = 11) and amicrofilaremic (Mp MF-; n = 10) individuals were analyzed for frequencies (%) of (**A**) CD4^+^CD127^+^ expressing CD25^high^ Tregs, (**B**) CD4+α/βTCR^+^ expressing CD49b and LAG3 Tr1 cells and (**C**) CD19^+^CD24^high^CD38^high^ expressing CD1d^high^ Bregs. Graphs show box whiskers with median, interquartile ranges and outliers. Statistical significances between the indicated groups were obtained using the Mann-Whitney-U-test.

Filarial nematodes modulate the host immune system through various strategies but the induction of regulatory B and T cells (Bregs and Tregs, respectively) are crucial to orchestrate the reduction of immune responses [1, 28, 31]. Indeed, frequencies of the Treg subset CD4^+^CD127^-^CD25^high^ [32] were significantly enhanced in Mp MF+ individuals (Fig 7A and S7A Fig). In contrast, type 1 regulatory T (Tr1) cells are characterized by the expression of CD49b and LAG3 [33] and the secretion of IL-10 and TGF-β have been shown to be important during onchocerciasis [31]. Thus, we also analysed the frequencies of Tr1 cells according to the applied gating strategy (S7B Fig). Surprisingly, frequencies of CD4+α/βTCR^+^CD49b^+^LAG3^+^ Tr1 cells were reduced in Mp MF+ compared to Mp MF- individuals (Fig 7B), suggesting that *M*. *perstans* differentially activates and/or inhibits regulatory T cell populations to orchestrate the modulation of the host immune system. In contrast, regulatory B cell (CD19^+^CD24^high^CD38^high^CD1d^high^) frequencies were strongly increased in the Mp MF+ cohort (Fig 7C and S8 Fig), highlighting that a variety of different regulatory cell populations are induced during *M*. *perstans* infection.

In summary, flow cytometry analysis of peripheral whole blood revealed a distinct immune cell composition in *M*. *perstans*-microfilaremic individuals characterized by increased frequencies of Th2, NK and regulatory B and T cells concomitant with decreased Tr1 cells.

## Discussion

Immunological research on *M*. *perstans* has been restricted due to the lack of appropriate antigen since adult worms reside in cavities of the human host and are therefore difficult to obtain [8–10]. *M*. *perstans* is perhaps the most competent of the filarial species in this regard, since infections result only in mild symptoms [8, 9]. Thus, our initial immune profiling of *M*. *perstans* microfilaremic individuals showed a broad spectrum of dampened immune responses, highlighting their skill at evading host immune responses. Until now, little is known about the potential *M*. *perstans* immune-modulatory mechanisms despite high infection rates and MF burden. To investigate *M*. *perstans*-driven immune responses, we used for the first time, *M*. *perstans* worm antigen extract that was free of bacterial contamination and could elicit *M*. *perstans*-specific responses. Indeed, we showed that re-stimulation of peripheral blood cells with worm antigen elicits strong IFN-γ, IL-17A and IL-13 responses from *M*. *perstans* microfilaremic individuals. Since cytokines were not detected in supernatants of cell cultures from healthy European NEN individuals that were never exposed to *M*. *perstans*, we conclude that the observed responses from *M*. *perstans-*microfilaremic (Mp MF+) individuals are worm specific. Interestingly, the immune responses from *M*. *perstans*-induced cell cultures of Mp MF+ individuals did not reflect their systemic serum cytokine profiles. For example, systemic IFN-γ, IL-13, IL-10 and IL-17A responses were all lower in infected individuals when compared to levels in amicrofilaremic (Mp MF-) individuals. Strong IL-17A and IL-17A/IL-4 responses in *W*. *bancrofti* and *O*. *volvulus-*infected individuals have respectively been associated with patients presenting different forms of associated pathology [34–36]. However, our previous studies on *O*. *volvulus* also revealed differing systemic and re-stimulated immune responses. In that study systemic IL-5 levels were significantly higher in microfilaremic (MF+) individuals when compared to amicrofilaremic (aMF) individuals but re-stimulation of isolated PBMC with *O*. *volvulus* antigen showed the opposite picture [21]. Therefore, contrary responses may also reflect the immune-activating capacity of adult worms and MF in the host. In addition, detection of *M*. *perstans* currently (in the absence of a circulating antigen test) depends on the presence of MF in the peripheral blood. Nevertheless, as mentioned above, infections of humans with *O*. *volvulus* or *W*. *bancrofti* as well as of rodents with the filaria *Litomosoides sigmodontis*, which shares various biological features with filariae that parasitize man, can be latent, i.e. not show skin or blood MF [21, 26, 29, 37]. Thus, it is important that further studies determine whether *M*. *perstans* MF- latent infections occur since this could provide an explanation for contradictory immune profiles in Mp MF- individuals. In future studies it will be interesting to analyse the responses of *M*. *perstans* MF- as well as *O*. *volvulus* and/or *Loa loa*-infected individuals to *M*. *perstans* worm antigen extract. It is likely that stimulated cell cultures from such infected individuals will respond to *M*. *perstans* antigen since previous studies from our group on onchocerciasis have shown that *O*. *volvulus*-infected individuals responded to *Brugia malayi* worm extract under similar condition [21]. Due to the low antigen yield, only a small number of NEN could be included in the current *in vitro* re-stimulation assays. However, as a proof of principle study, these data still provide important information about the ability of our *in vitro* generated worm antigen to elicit *M*. *perstans*-specific immune responses, especially since cell cultures from NEN failed to elicit any cytokine responses. Further studies should also focus on responses to excretory/secretory products from the cultured worms to obtain a more detailed analysis. In addition, in the present study, patient numbers differ between the analyses of immune responses and cell populations due to the lack of serum, blood or *M*. *perstans* antigen in some patients, but individual parameters like age distribution or MF load were comparable between the individuals. Importantly, the differences observed in the analysed immune parameters were not strongly influenced by gender, since additional analysis of men only did not alter most cytokine, chemokine and immunoglobulin secretion patterns. The fact that only one woman was positive for *M*. *perstans* MF might suggest that women are more resistant to *M*. *perstans* infection than men in this endemic area. Indeed, previous studies have shown that women are more resistant to *Wuchereria bancrofti* and *Onchocerca volvulus* infection [38, 39] and an epidemiological survey in southern Cameroon revealed that more men were infected with *M*. *perstans* than women [40]. Thus, further studies need to include more individuals and should also identify the level of cross-reactivity of *M*. *perstans* antigen with other filarial-derived antigens since our previous study on *O*. *volvulus*-infected individuals only detected subtle differences in immune responses when the specific *O*. *volvulus*-derived antigen was applied [21].

Analysis of systemic chemokine and cytokine levels revealed that only MIP-1β was significantly elevated in the Mp MF+ cohort when compared to Mp MF- individuals and levels positively correlated with MF load. All other chemokines (IL-8, RANTES) or proinflammatory (IL-6, TNF-α), Th1 (IFN-γ, IL-12p70) and Th2/Th17 (IL-4, IL-5, IL-10, IL-13, IL-17A) cytokines were equal or significantly higher in Mp MF- individuals. The chemokine MIP-1β, also known as CCL4, is a chemoattractant for a variety of immune cells [41], promotes macrophage-mediated Th1 proinflammatory responses [42] and is the major HIV-suppressive factor produced by CD8^+^ T cells [43]. Interestingly, studies in *O*. *volvulus*-infected individuals showed that systemic MIP-1β levels were low and similar to healthy controls, but upon ivermectin treatment levels began to rise 6 months post-therapy [44]. The functional role of MIP-1β during *M*. *perstans* infection requires further investigation and moreover its role or presence in other filarial infections. Nevertheless, the reduction of systemic cytokine and chemokine levels were also reflected in the immune cell composition of peripheral whole blood since Mp MF+ individuals showed increased frequencies of Th2 (CD4^+^CRTH2^+^) and Treg (CD4^+^CD127^-^CD25^high^) subsets, but reduced type 1 regulatory T (Tr1) cells (CD4+α/βTCR^+^CD49b^+^LAG3^+^). In addition, several studies have shown that Treg populations including Tr1 like cells are an important source of the regulatory cytokines IL-10 and TGF-β and might be crucial to orchestrate immune-modulatory mechanisms in filarial-infected individuals [28, 45–48]. Thus, further studies need to be performed to decipher regulatory T cell populations more in detail and determine if the CD49b^+^LAG3^+^ Tr1 cell population secretes IL-10 and TGF-β and moreover, if induced and/or natural Treg subsets appear in *M*. *perstans* infected individuals. In contrast, CD19^+^CD24^high^CD38^high^CD1d^high^ regulatory B (Breg) cells were significantly increased in the Mp MF+ cohort, showing that besides regulatory T cell subsets other immune cell populations are crucial for the immune-modulation during *M*. *perstans* infection. Little is known about Breg populations in helminth infection and so far only a few studies have shown that Breg subsets which produce IL-10 were increased in schistosome-infected individuals and might be important to modulate host immunity [49–51]. However, for the first time, this study shows that Breg populations are increased in *M*. *perstans*-infected individuals, suggesting an important role for these immune-modulating cells during mansonellosis and thus, further investigations should unravel the role of Breg cell subsets and the corresponding IL-10 secretion during filarial infection. Nevertheless, the broad immune-regulatory phenotype in Mp MF+ individuals was further highlighted by the increased expression of the regulatory effector molecule PD-1 [52] on CD8^+^ cytotoxic T cells. In addition, CTLA-4 expression was also shown to be up-regulated in filarial infected individuals [53–55] but within this study expression levels of CTLA-4 on CD4^+^ and CD8^+^ T cells was equal between Mp MF- and Mp MF+ individuals, suggesting that *M*. *perstans* specifically induce immunomodulatory mechanisms which might be different from those observed in onchocerciasis or lymphatic filariasis. In addition, the increased frequency of natural killer cells (CD3^-^CD16^+^CD56^+^) is another interesting feature of *M*. *perstans*-infected individuals indicating that besides the modulation of the adaptive immune system, *M*. *perstans* also influences innate immune cell subsets as well as innate immune responses. However, additional flow cytometry analysis needs to be performed to decipher other innate immune cell population like macrophages, eosinophils or neutrophils.

Since we obtained *M*. *perstans* worm antigen extract, we were also able to assess *M*. *perstans*-specific immunoglobulins. Due to the limited *M*. *perstans* worm antigen extract we chose to concentrate on IgE and IgG4 levels but future studies should also investigate differences in other Ig classes and subtypes. We show now, for the first time, that *M*. *perstans*-specific IgG4 levels are also associated with patent *M*. *perstans* infection. This finding further confirms the association of enhanced IgG4 expression with a regulated immune system observed in patients infected with *W*. *bancrofti* or *O*. *volvulus* [21, 26, 56]. In addition, total IgE and IgG4 levels were also enhanced by tendency in Mp MF+ compared to Mp MF- individuals which supports a recent study in Gabon showing higher total IgE levels in *M*. *perstans*-infected (MF+) individuals [57]. Although *M*. *perstans*-specific IgE levels were low this may complement our systemic immune profiling which revealed how well *M*. *perstans* appears to avoids the host's immune system. However, the detected *M*. *perstans*-specific IgE levels in Mp MF- individuals could also be due to the possibility that Mp MF- individuals were previously exposed to *M*. *perstans* or harbour adult worms which do not produce MF. In addition, individuals from both cohorts were positive for IgG4 antibodies against the OV16 antigen, showing that exposure to *O*. *volvulus* or occult infections are common in South-West Cameroon. However, only RANTES secretion was associated with the *O*. *volvulus*-specific IgG4 diagnostic marker highlighting that the observed differences in cytokine/chemokine levels and immune cell frequencies were caused by *M*. *perstans* infection and not due to exposure or occult *O*. *volvulus* infection. Indeed, Cooper *et al*. showed that RANTES is essential for eosinophil recruitment and cellular killing of *O*. *volvulus* MF [58, 59], suggesting that previous *O*. *volvulus* infection and thus circulation of *O*. *volvulus*-specific IgG4 antibodies might influence RANTES signalling to maintain protection against filarial re-infection.

In summary, within this study, we aimed to perform an initial immunologically characterization of *M*. *perstans* infected individuals from the South-West region of Cameroon by excluding active co-infections with other filariae. This study provides an initial overview on the systemic filarial-specific immune responses and immune cell composition in *M*. *perstans*-infected individuals. Until now, findings were based on studies were patients were co-infected with other parasites like *O*. *volvulus*, *W*. *bancrofti*, *Plasmodium falciparum*, *Entamoeba histolytica/E*. *dispar*, *Schistosoma mansoni/S*. *haematobium* [11–15]. We found that, with the exception of MIP-1β and IL-10, serum cytokine and chemokine levels of *M*. *perstans*-microfilaremic individuals were all reduced. This finding was reflected by the immune cell composition in peripheral blood from Mp MF+ individuals which revealed increased Th2, NK, regulatory B and regulatory T cell population’s concomitant with decreased Tr1 cells. For the first time, *M*. *perstans* antigen-specific cultures of peripheral cells however showed elevated IFN-γ, IL-10, IL-13 and IL-17A responses and moreover antigen-specific IgG4 levels were enhanced in *M*. *perstans* infected individuals. Collectively, these findings immunological characterize *M*. *perstans*-infected individuals which are MF+ for the first time and provide insight into the potential *M*. *perstans*-driven immunomodulatory mechanisms which might be responsible for the increased susceptibility and worsened disease course of HIV, tuberculosis (TB) and malaria [60–62] and the lower efficacy of Bacillus Calmette–Guérin (BCG) vaccination against TB [63] in regions where *M*. *perstans* is prevalent.

## Supporting information

S1 Table Characteristics of the study population for analysis of systemic cytokine and chemokine levels. (PDF)

S2 Table Characteristics of study population for the analysis of serum immunoglobulin levels. (PDF)

S3 Table Characteristics of study population for the *M*. *perstans*-specific re-stimulation assays. (PDF)

S4 Table Characteristics of study population for flow cytometry. (PDF)

S1 Fig Systemic cytokine secretion patterns remain unchanged upon exclusion of women. Sera from *M*. *perstans*-microfilaremic (Mp MF+, n = 10) and amicrofilaremic (Mp MF-; n = 15) male participants were analyzed for the levels of (**A**) IFN-γ, (**B**) IL-5, (**C**) IL-13, (**D**) IL-4, (**E**) IL-10 and (**F**) IL-17A using luminex technology. Graphs show box whiskers with median, interquartile ranges and outliers. Statistical significances between the indicated groups were obtained using the Mann-Whitney-U-tests.
(TIF)

S2 Fig Elevated systemic levels of MIP-1β levels in *M*. *perstans* microfilaremic men. Sera from *M*. *perstans* microfilaremic (Mp MF+, n = 10) and amicrofilaremic (Mp MF-; n = 15) male participants were analyzed for the levels of (**A**) IL-6, (**B**) TNF-α, (**C**) IL-12p70, (**D**) IL-8, (**E**) RANTES and (**F**) MIP-1β using luminex technology. Graphs show box whiskers with median, interquartile ranges and outliers. Statistical significances between the indicated groups were obtained using the Mann-Whitney-U-tests.
(TIF)

S3 Fig *M*. *perstans* microfilaremic men present elevated filarial-specific IgG4 in sera. Sera from and *M*. *perstans* microfilaremic (Mp MF+, n = 10) and amicrofilaremic (Mp MF-; n = 6) male participants were analyzed for total and *M*. *perstans*-specific immunoglobulin levels. (**A**) total IgE (μg/ml) (**B**) total IgG4 (μg/ml), (**C**) ratio of total IgE/IgG4, (**D**) *M*. *perstans*-specific IgE (OD), (**E**) *M*. *perstans*-specific IgG4 (OD) and (**F**) ratio of *M*. *perstans*-specific IgG4/IgE. Graphs show box whiskers with median, interquartile ranges and outliers. Statistical significances between the indicated groups were obtained using the Mann-Whitney-U-test.
(TIF)

S4 Fig Increased CD8a^+^PD-1^+^ cytotoxic T cell frequencies in peripheral blood of *M*. *perstans* microfilaremic individuals. Using flow cytometry, peripheral whole blood cells from *M*. *perstans* microfilaremic (Mp MF+; n = 11) and amicrofilaremic (Mp MF-; n = 10) individuals were analyzed for frequencies (%) of (**A**) CD8a T cells expressing either (**B**) CTLA-4 or (**C**) PD-1. Graphs show box whiskers with median, interquartile ranges and outliers. Statistical significances between the indicated groups were obtained using the Mann-Whitney-U-test.
(TIF)

S5 Fig Gating strategy for CD4^+^ and CD8a^+^ T cell populations. Peripheral blood cells were stained with fluorophore-conjugated anti-human CD4, CD8a, CXCR3, CRTH2, CD161, CTLA-4 and PD-1 monoclonal antibodies and frequencies of (**A**) CD4^+^ T cells or (**B**) CD8a^+^ T cell populations were analysed according to the presented gating strategy.
(TIF)

S6 Fig Gating strategy for NKT and NK cells. Peripheral blood cells were stained with fluorophore-conjugated anti-human CD3, CD16 and CD56 monoclonal antibodies and frequencies of (**A**) CD3^+^CD16^+^CD56^+^ NKT or (**B**) CD3^-^CD16^+^CD56^+^ NK cells were analysed according to the presented gating strategy.
(TIF)

S7 Fig Gating strategy for Treg and Tr1 cells. Peripheral blood cells were stained with fluorophore-conjugated anti-human CD4, CD25, CD49b, CD127, α/βTCR and LAG3 monoclonal antibodies and frequencies of (**A**) CD4^+^CD127^-^CD25^high^ Tregs and (**B**) CD4+α/βTCR^+^ CD49b^+^LAG3^+^ Tr1 cells were analysed according to the presented gating strategy.
(TIF)

S8 Fig Gating strategy for Bregs. Peripheral blood cells were stained with fluorophore-conjugated anti-human CD1d, CD19, CD24 and CD38 monoclonal antibodies and frequencies of CD19^+^CD24^high^CD38^high^CD1d^high^ Bregs were analysed according to the presented gating strategy.
(TIF)
