# Supplementary material for: Mansonella perstans microfilaremic individuals are characterized by enhanced type 2 helper T and regulatory T and B cell subsets and dampened systemic innate and adaptive immune responses
Source: PLoS Negl Trop Dis. 2018 Jan 11;12(1):e0006184. doi: 10.1371/journal.pntd.0006184 (PMC5783424; doi:10.1371/journal.pntd.0006184)
Supplement: S2 Table — (PDF) [file pntd.0006184.s002.pdf]

S4 Table: Characteristics of study population for the analysis of serum immunoglobulin levels

Characteristics	Mp MF+	Mp MF-
Total sample size (n)	11	6
Mean age (range) [years]	36.5 (26-64)	29.3 (10-38)
Median age (range) [years]	37 (26-64)	33 (10-38)
Gender [Female:Male]	1:10	0:6
Health district	Konye, Kumba, Tombel,	Konye, Kumba, Tombel
Community	Baduma, Matondo, Mbalangui, Mbule	Baduma, Matondo, Mbalangui, Mbule
Mean of microfilaria count (range) [MF/ml]	75.4 (1-364)	0
Median of microfilaria count (range) [MF/ml]	53 (1-364)	0
Number of Ov16-specific IgG4 positive individuals	10 (out of 11)	2 (out of 3)
Number of individuals positive for STHs	1 (*Ascaris lumbricoides*)	0
