# Supplementary material for: Mansonella perstans microfilaremic individuals are characterized by enhanced type 2 helper T and regulatory T and B cell subsets and dampened systemic innate and adaptive immune responses
Source: PLoS Negl Trop Dis. 2018 Jan 11;12(1):e0006184. doi: 10.1371/journal.pntd.0006184 (PMC5783424; doi:10.1371/journal.pntd.0006184)
Supplement: S3 Table — (PDF) [file pntd.0006184.s003.pdf]

S6 Table: Characteristics of study population for the *M. perstans*-specific re-stimulation assays

Characteristics	Mp MF+	NEN
Total sample size (n)	9	4
Mean age (range) [years]	36.8 (26-60)	44.5 (34-55)
Median age (range) [years]	38 (26-60)	44.5 (34-55)
Gender [Female:Male]	0:9	0:4
Health district	Tombel	Bonn
Community	Mbule	Bonn
Mean of microfilaria count (range) [MF/ml]	117.8 (11-364)	0
Median of microfilaria count (range) [MF/ml]	102 (11-364)	0
Number of Ov16-specific IgG4 positive individuals	8 (out of 9)	0
Number of individuals positive for STHs	1 (*Ascaris lumbricoides*)<br>1 (*Trichuris trichiura*)	0
