# Supplementary material for: Mansonella perstans microfilaremic individuals are characterized by enhanced type 2 helper T and regulatory T and B cell subsets and dampened systemic innate and adaptive immune responses
Source: PLoS Negl Trop Dis. 2018 Jan 11;12(1):e0006184. doi: 10.1371/journal.pntd.0006184 (PMC5783424; doi:10.1371/journal.pntd.0006184)
Supplement: S4 Table — (PDF) [file pntd.0006184.s004.pdf]

S7 Table: Characteristics of study population for flow cytometry analysis

Characteristics	Mp MF+	Mp MF-
Total sample size (n)	11	10
Mean age (range) [years]	36.9 (26-60)	30.9 (23-43)
Median age (range) [years]	38 (26-60)	28.5 (23-43)
Gender [Female:Male]	0:11	0:10
Health district	Tombel	Buea, Tombel
Community	Mbule	Bomaka, Mbule, Mile-14, Minicokuette, Molyko, Sampit
Mean of microfilaria count (range) [MF/ml]	207.9 (11-745)	0
Median of microfilaria count (range) [MF/ml]	109 (11-745)	0
Number of Ov16-specific IgG4 positive individuals	8 (out of 10)	0 (out of 9)
Number of individuals positive for STHs	2 (*Ascaris lumbricoides*)<br>1 (*Trichuris trichiura*)	0
