# Supplementary figures and images for: Mansonella perstans microfilaremic individuals are characterized by enhanced type 2 helper T and regulatory T and B cell subsets and dampened systemic innate and adaptive immune responses
Source: PLoS Negl Trop Dis. 2018 Jan 11;12(1):e0006184. doi: 10.1371/journal.pntd.0006184 (PMC5783424; doi:10.1371/journal.pntd.0006184)

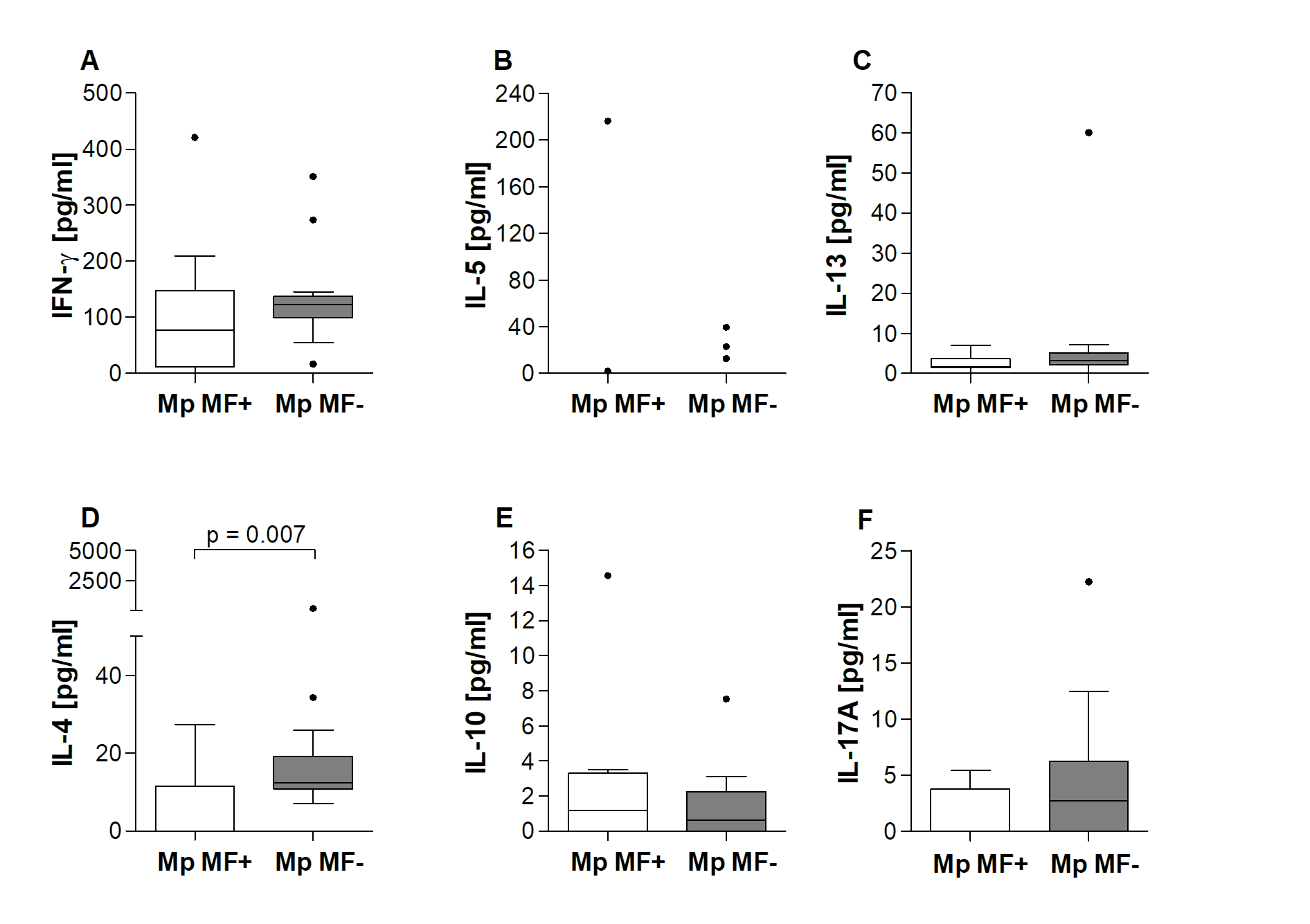

Supplement: S1 Fig — Sera from M. perstans-microfilaremic (Mp MF+, n = 10) and amicrofilaremic (Mp MF-; n = 15) male participants were analyzed for the levels of (A) IFN-γ, (B) IL-5, (C) IL-13, (D) IL-4, (E) IL-10 and (F) IL-17A using luminex technology. Graphs show box whiskers with median, interquartile ranges and outliers. Statistical significances between the indicated groups were obtained using the Mann-Whitney-U-tests. (TIF) [file pntd.0006184.s005.tif]

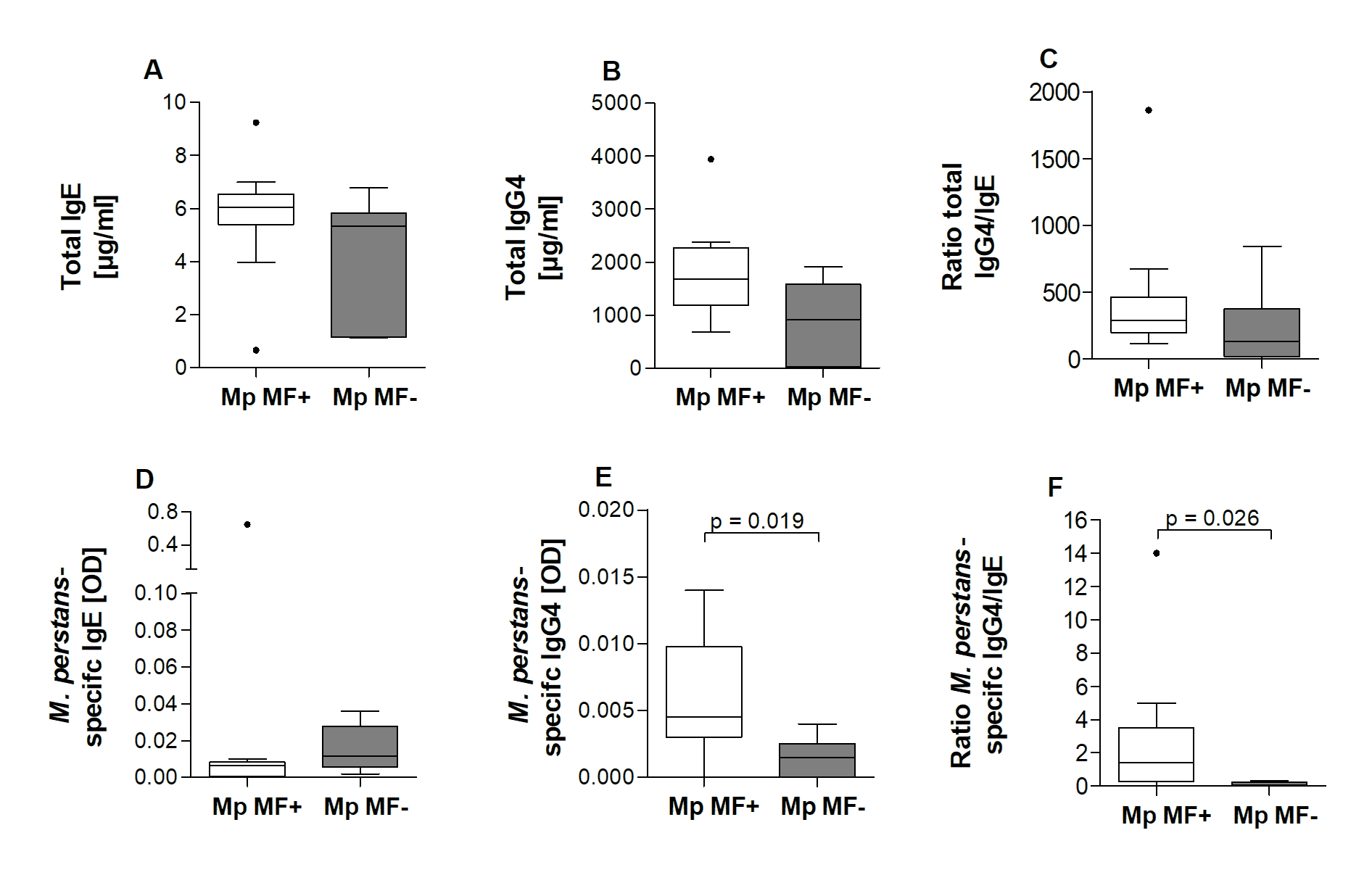

Supplement: S2 Fig — Sera from M. perstans microfilaremic (Mp MF+, n = 10) and amicrofilaremic (Mp MF-; n = 15) male participants were analyzed for the levels of (A) IL-6, (B) TNF-α, (C) IL-12p70, (D) IL-8, (E) RANTES and (F) MIP-1β using luminex technology. Graphs show box whiskers with median, interquartile ranges and outliers. Statistical significances between the indicated groups were obtained using the Mann-Whitney-U-tests. (TIF) [file pntd.0006184.s006.tif]

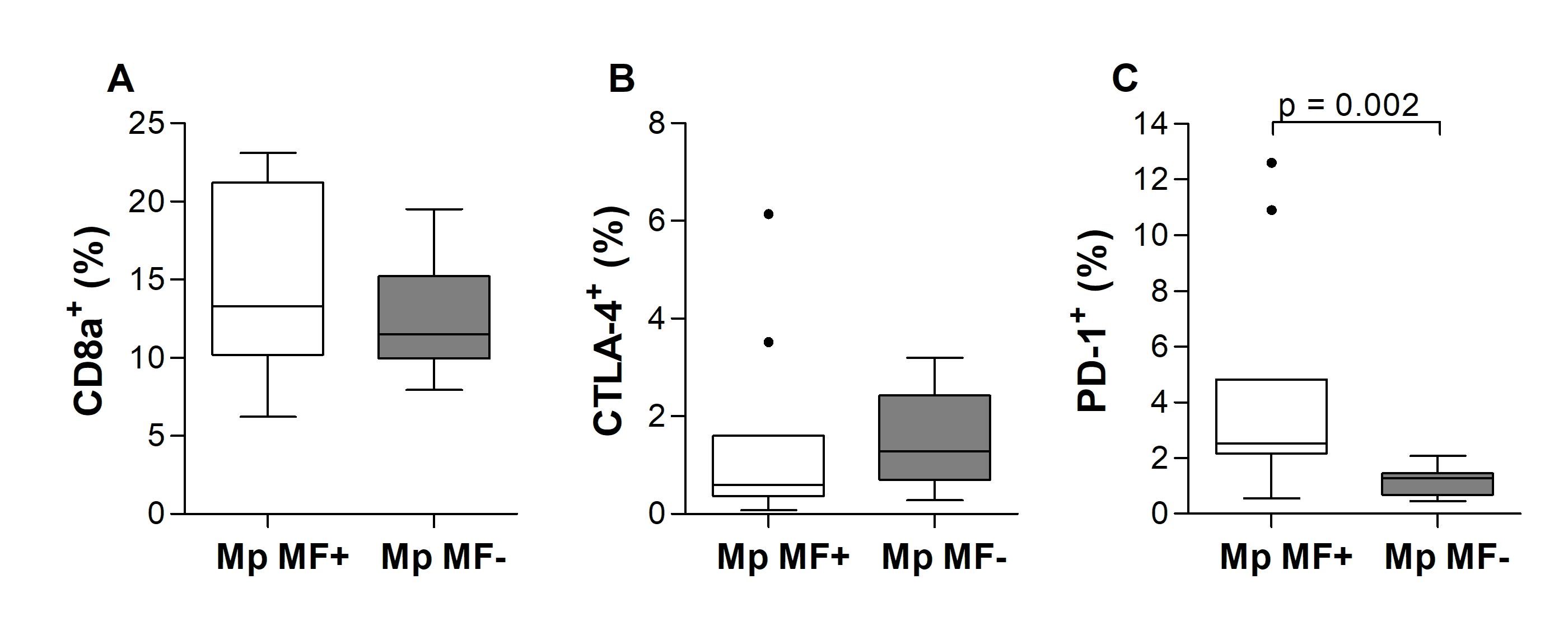

Supplement: S4 Fig — Using flow cytometry, peripheral whole blood cells from M. perstans microfilaremic (Mp MF+; n = 11) and amicrofilaremic (Mp MF-; n = 10) individuals were analyzed for frequencies (%) of (A) CD8a T cells expressing either (B) CTLA-4 or (C) PD-1. Graphs show box whiskers with median, interquartile ranges and outliers. Statistical significances between the indicated groups were obtained using the Mann-Whitney-U-test. (TIF) [file pntd.0006184.s008.tif]

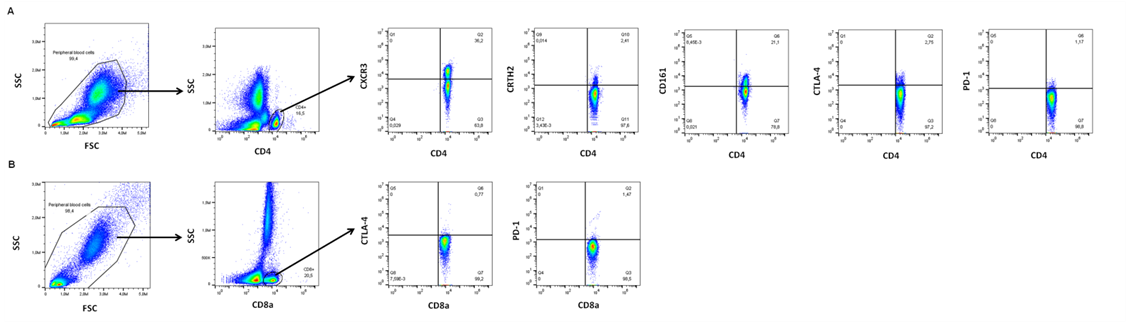

Supplement: S5 Fig — Peripheral blood cells were stained with fluorophore-conjugated anti-human CD4, CD8a, CXCR3, CRTH2, CD161, CTLA-4 and PD-1 monoclonal antibodies and frequencies of (A) CD4+ T cells or (B) CD8a+ T cell populations were analysed according to the presented gating strategy. (TIF) [file pntd.0006184.s009.tif]

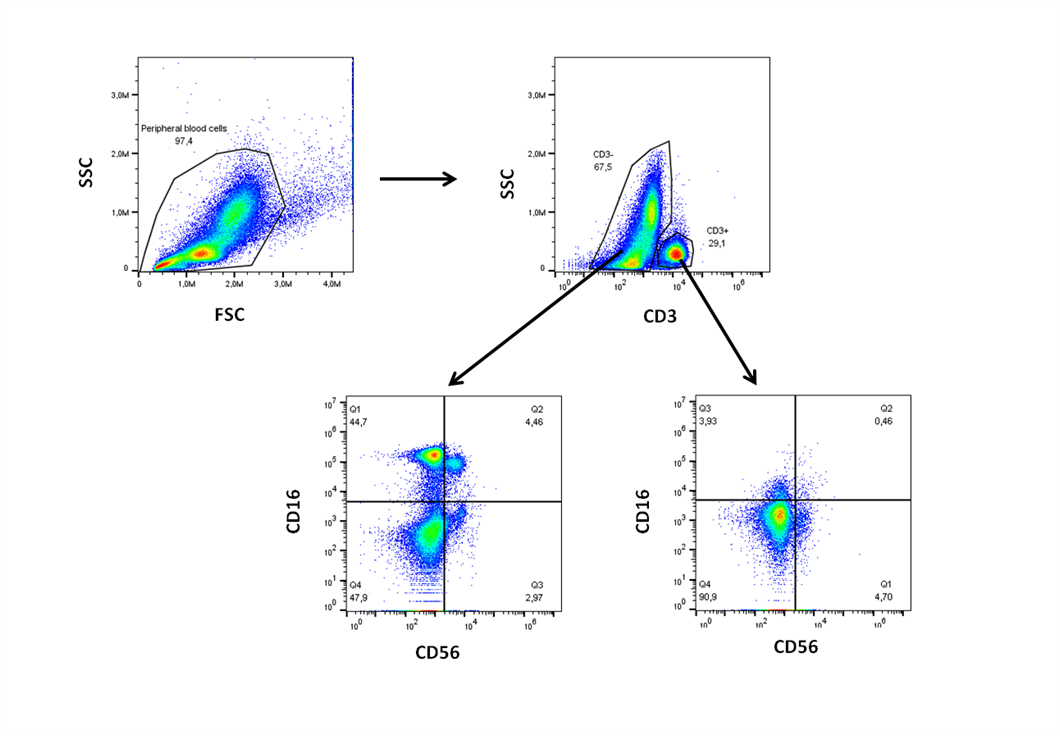

Supplement: S6 Fig — Peripheral blood cells were stained with fluorophore-conjugated anti-human CD3, CD16 and CD56 monoclonal antibodies and frequencies of (A) CD3+CD16+CD56+ NKT or (B) CD3-CD16+CD56+ NK cells were analysed according to the presented gating strategy. (TIF) [file pntd.0006184.s010.tif]

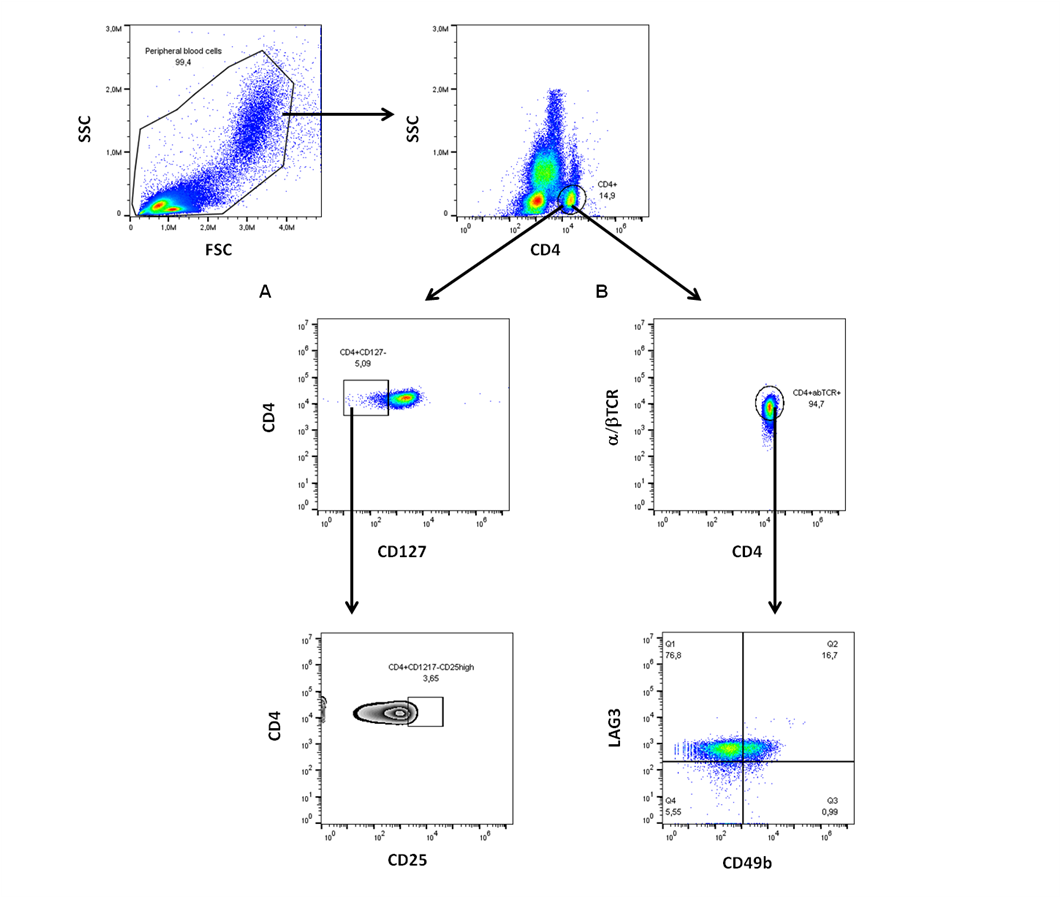

Supplement: S7 Fig — Peripheral blood cells were stained with fluorophore-conjugated anti-human CD4, CD25, CD49b, CD127, α/βTCR and LAG3 monoclonal antibodies and frequencies of (A) CD4+CD127-CD25high Tregs and (B) CD4+α/βTCR+ CD49b+LAG3+ Tr1 cells were analysed according to the presented gating strategy. (TIF) [file pntd.0006184.s011.tif]

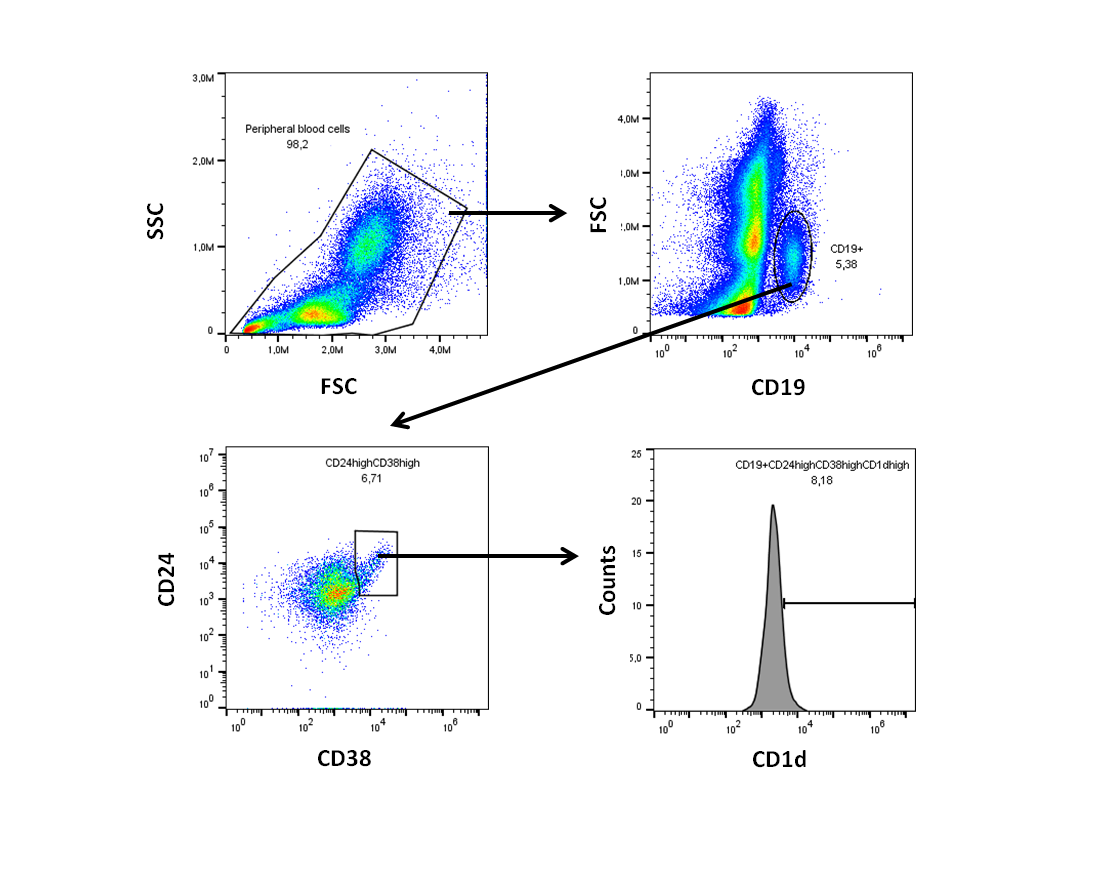

Supplement: S8 Fig — Peripheral blood cells were stained with fluorophore-conjugated anti-human CD1d, CD19, CD24 and CD38 monoclonal antibodies and frequencies of CD19+CD24highCD38highCD1dhigh Bregs were analysed according to the presented gating strategy. (TIF) [file pntd.0006184.s012.tif]
